# Postmarket Clinical Evidence for High-Risk Therapeutic Medical Devices Receiving Food and Drug Administration Premarket Approval in 2010 and 2011

**DOI:** 10.1001/jamanetworkopen.2020.14496

**Published:** 2020-08-28

**Authors:** Vinay K. Rathi, Harlan M. Krumholz, Frederick A. Masoudi, Joseph S. Ross

**Affiliations:** 1 Department of Otolaryngology, Massachusetts Eye and Ear, Boston; 2 Section of Cardiovascular Medicine, Department of Internal Medicine, Yale University School of Medicine, New Haven, Connecticut; 3 Department of Health Policy and Management, Yale University School of Public Health, New Haven, Connecticut; 4 Center for Outcomes Research and Evaluation, Yale-New Haven Hospital, New Haven, Connecticut; 5 Division of Cardiology, University of Colorado Anschutz Medical Campus, Aurora; 6 Colorado Cardiovascular Outcomes Research Consortium, Denver; 7 Section of General Internal Medicine, Department of Internal Medicine, Yale University School of Medicine, New Haven, Connecticut

## Abstract

This cross-sectional study provides a 5-year update on the status and availability of postmarket evidence for high-risk medical devices that received FDA premarket approval in 2010 and 2011.

## Introduction

The US Food and Drug Administration (FDA) approves high-risk medical devices primarily through the premarket approval pathway, which requires clinical evidence assuring device safety and effectiveness. By law, the FDA stipulates only the minimum premarket evidence necessary,^1^ which typically includes 1 feasibility study and 1 pivotal study.^2^ However, the FDA may require manufacturers to conduct postmarket studies to generate complementary performance data (eg, characterizations of long-term outcomes). In addition, manufacturers and independent investigators may conduct postmarket studies to answer other important questions (eg, benefit-risk assessment for new indications or certain populations).

In 2015, we reported that 13% of all studies initiated after FDA premarket approval were completed within 3 to 5 years, while 75% were ongoing.^2^ Now, 5 years later, we sought to determine whether additional studies were completed and reported.

## Methods

This cross-sectional study followed the Strengthening the Reporting of Observational Studies in Epidemiology (STROBE) reporting guideline. The Human Research Protections Program at Massachusetts Eye and Ear determined that review and informed consent were not required, because this study analyzed publicly available data and did not involve study participants. We conducted a cross-sectional analysis of all previously identified postmarket studies evaluating novel high-risk therapeutic devices approved in 2010 and 2011.^2^ For each study, we searched ClinicalTrials.gov to determine the (1) initiation date, (2) current status (ie, completed, ongoing, or terminated or unknown), (3) earliest date of final results reporting, and (4) whether interim study results were reported. We then searched PubMed using National Clinical Trial identifiers to identify publications.

We evaluated the published final results of all previously identified postmarket multiarmed studies, which assess comparative safety and effectiveness, of high-risk medical devices. For each study, we categorized the design type (ie, superiority, noninferiority, or equivalence) and reviewed the abstract to determine primary end point results at follow-up completion and device-related safety concerns.

We used descriptive statistics to summarize all information. Searches were completed between December 18, 2019, and January 24, 2020. All analyses were performed in Excel version 2016 (Microsoft) from January 24 to June 8, 2020.

## Results

Our original report included 204 postmarket studies evaluating 28 high-risk therapeutic medical devices as of December 16, 2014.^2^ Of these devices, 21 (75.0%) were implantable, 9 (28.1%) were life sustaining, and 15 (53.6%) were for cardiovascular conditions.^2^ After 5 more years of follow-up, 113 postmarket studies (55.4%) were completed (Figure), including 25 of 33 FDA-required studies (75.8%), while 38 postmarket studies (18.6%) were ongoing and 53 postmarket studies (26.0%) were terminated or had an unknown status. Final results were reported (via Clinical Trials.gov or publications) for 75 of the completed studies (36.8% of the original 204 postmarket studies). Median (interquartile range) time from study initiation to reporting of final results was 5.1 (3.6-7.1) years. Interim results were reported for an additional 15 ongoing or terminated/unknown studies (16.5%).

**Figure.**
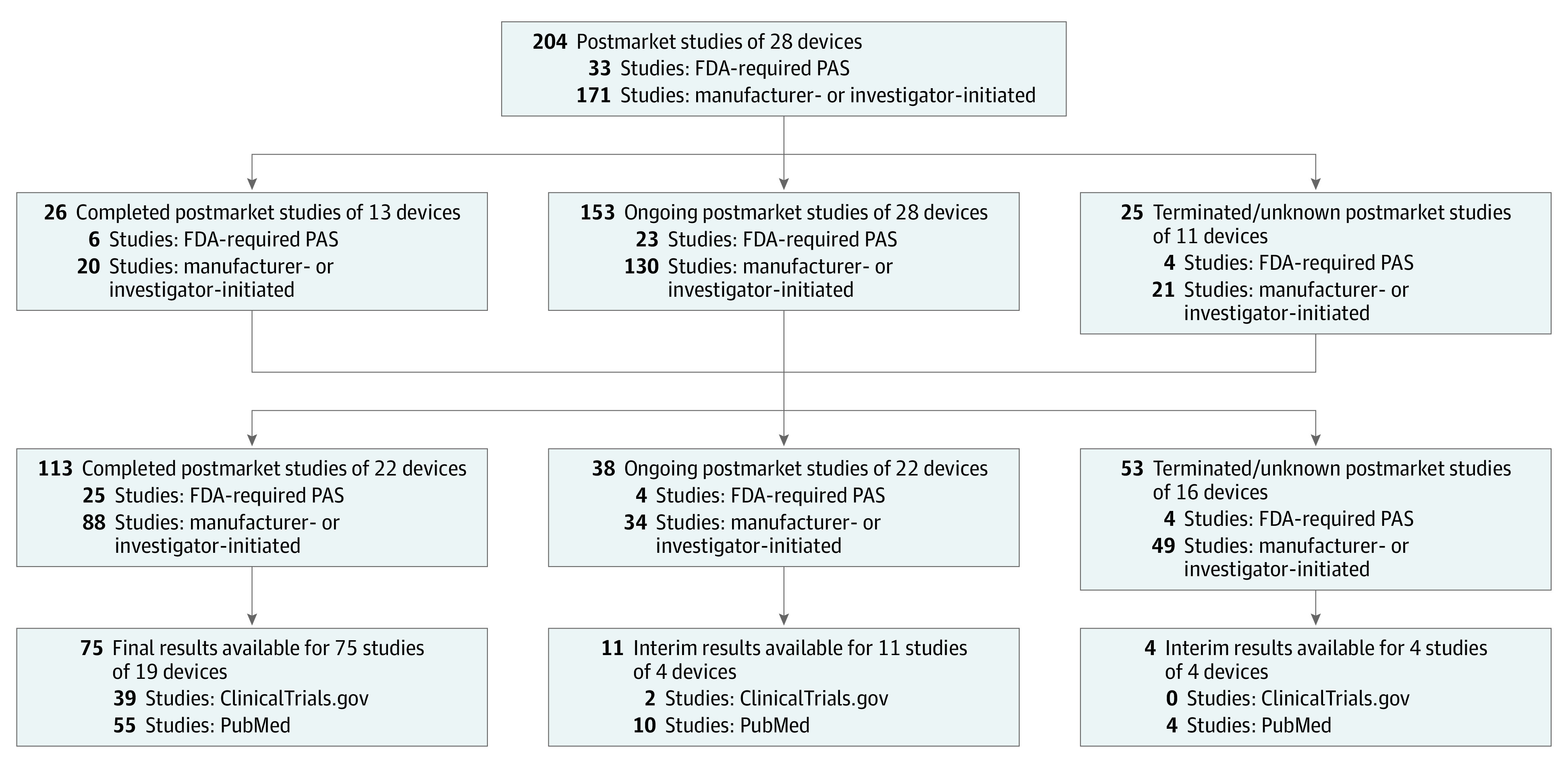
Updated Status and Results Availability for Postmarket Studies of High-Risk Therapeutic Devices Receiving Food and Drug Administration (FDA) Premarket Approval in 2010 and 2011 Updated study status as of January 2020. Terminated/unknown status includes studies listed as “terminated,” “unknown,” or “withdrawn” on ClinicalTrials.gov. Results availability was not mutually exclusive for ClinicalTrials.gov and PubMed. Some studies were completed after they were classified as unknown in December 2014 because of trial sponsor inactivity on ClinicalTrials.gov. Results were not reported for some studies classified as completed on ClinicalTrials.gov. Excludes 28 completed studies for which interim results were additionally available, including 7 completed studies without publicly reported final results. PAS indicates postapproval studies.

Among 33 completed multiarmed studies with published final results, 22 (66.7%) used a superiority design; of these, 12 (54.5%) failed to demonstrate superiority, including 2 (9.1%) that found inferiority. The abstracts of 4 studies (12.1%) described device-related safety concerns, such as increased risk of phrenic nerve palsy.

## Discussion

Among postmarket studies evaluating high-risk therapeutic medical devices approved in 2010 and 2011, approximately one-third were completed and reported final results via Clinical Trials.gov or journal publication within 8 to 10 years after FDA approval. More than 40% were ongoing or were terminated or had unknown status.

When results were disseminated, postmarket studies yielded important insights. For instance, a large, multicenter, double-blind randomized clinical trial^3^ revealed that intra-articular hyaluronic acid injection was not superior to placebo in reducing knee osteoarthritis pain. In another study,^4^ investigators halted evaluation of an intracranial aneurysm flow diverter because treatment resulted in high rates of death and dependency.

Our study has limitations. First, we did not enhance our original sample to include postmarket studies initiated after December 2014, potentially underestimating the number of completed and reported postmarket studies. However, our study sample included all postmarket studies initiated within the first few years after approval, which are likely to have the greatest potential to inform clinical adoption. Second, we restricted our analysis to high-risk devices; our findings are not generalizable to low- or moderate-risk devices, for which the FDA typically does not require premarket or postmarket clinical evidence.^5^

Our results suggest that patients and clinicians may have limited evidence available to inform treatment decisions about high-risk medical devices up to a decade after approval. Policy makers should consider measures to promote timely completion and reporting of postmarket studies. These approaches could include enforcing financial penalties for failure to report results on ClinicalTrials.gov or funding independent comparative effectiveness research using milestone payments linked to study progress and publication.
